# High Shear Stresses under Exercise Condition Destroy Circulating Tumor Cells in a Microfluidic System

**DOI:** 10.1038/srep39975

**Published:** 2017-01-05

**Authors:** Sagar Regmi, Afu Fu, Kathy Qian Luo

**Affiliations:** 1 School of Chemical and Biomedical Engineering, Nanyang Technological University, Singapore; 2 Faculty of Health Sciences, University of Macau, Taipa, Macau, China

## Abstract

Circulating tumor cells (CTCs) are the primary targets of cancer treatment as they cause distal metastasis. However, how CTCs response to exercise-induced high shear stress is largely unknown. To study the effects of hemodynamic microenvironment on CTCs, we designed a microfluidic circulatory system that produces exercise relevant shear stresses. We explore the effects of shear stresses on breast cancer cells with different metastatic abilities, cancer cells of ovarian, lung and leukemic origin. Three major findings were obtained. 1) High shear stress of 60 dynes/cm^2^ achievable during intensive exercise killed more CTCs than low shear stress of 15 dynes/cm^2^ present in human arteries at the resting state. 2) High shear stress caused necrosis in over 90% of CTCs within the first 4 h of circulation. More importantly, the CTCs that survived the first 4 h-circulation, underwent apoptosis during 16–24 h of post-circulation incubation. 3) Prolonged high shear stress treatment effectively reduced the viability of highly metastatic and drug resistant breast cancer cells. As high shear stress had much less damaging effects on leukemic cells mimicking the white blood cells, we propose that intensive exercise may be a good strategy for generating high shear stress that can destroy CTCs and prevent cancer metastasis.

Cancer metastasis is a major medical problem because it causes 90% of human cancer deaths^1^, thus the most effective way to save the life of cancer patients is to prevent metastasis. Metastasis occurs through a series of complicated steps including: 1) tumor cells depart from the primary tumor sites; 2) the cells undergo intravasation to enter the circulatory system^2^,^3^; 3) the cells travel in the bloodstream known as circulating tumor cells (CTCs); and 4) finally, the survived CTCs extravasate and form secondary tumors in different parts of the body^4^. As only the survived CTCs can become the initial metastatic tumor cells, destroying these CTCs represents a promising strategy to prevent metastasis^5^. Many studies have shown that CTCs can serve as a prognostic marker^6^ for patients with prostate, metastatic breast and colorectal cancer^7^. However, how to eliminate CTCs without damaging the blood cells remains a big challenge. Previously, systematic reviews and meta-analyses of randomized controlled trials suggested that physical exercise can benefit patients with HIV/AIDS^8^, coronary heart disease^9^ and cancer^10^. However, little is known about the effect of physical exercise on the viability of CTCs.

CTCs can potentially be destroyed in the bloodstream by several mechanisms including hemodynamic shear stress (SS), anoikis due to the detachment of the CTCs from the extracellular matrix, and immune-elimination^11^. Among them, hemodynamic SS is the main focus of this study because it has been reported that SS generated by the bloodstream can destroy cancer cells, rendering the metastatic process ineffective^2^,^12^. Previously, several studies have investigated the effects of SS on endothelial cells^13^,^14^,^15^, cardiovascular disease^16^, atherosclerosis^17^, etc. Recently, we also reported that physiological levels of SS could induce apoptosis in circulating breast cancer cells^18^. However, it is not well understood how high levels of SS achievable under intensive exercise conditions can affect CTCs, especially the ones with increased levels of malignancy.

To address this question, we have developed a bio-mimicking circulatory system that can produce a broader range of SS than the one reported in our previous study^18^. On average, hemodynamic SS is 15 dynes/cm^2^ in human arteries and 1–6 dynes/cm^2^ in veins at resting state^12^,^19^. During arm cycle exercise, the SS can increase to 60 dynes/cm^2^ in the femoral artery^20^. In a human body, the blood flows in a pulsatile manner^21^, hence we also mimicked this pulsatile mode in our microfluidic system^18^. We then compared the effects of low and high SS on a series of breast cancer cells with different metastatic abilities^18^, lung and ovarian cancer cells. The *in vitro* microfluidic circulatory system developed in this study circumvents a major obstacle in studying clinically isolated CTCs, i.e. the extremely low level of CTCs (1–5 cells/ml of patient’s blood sample^7^). Some of the breast cancer cells used in this study also stably expressed apoptotic sensor proteins which allow real-time detection of apoptosis^18^,^22^,^23^. By combining the three technologies including the microfluidic circulatory system, metastatic cell lines, and apoptotic sensor, we were able to closely examine how high SS generated during intensive exercise destroys CTCs.

## Results

### Design of a microfluidic system for generating a broad range of hemodynamic SS

**Figure 1:**
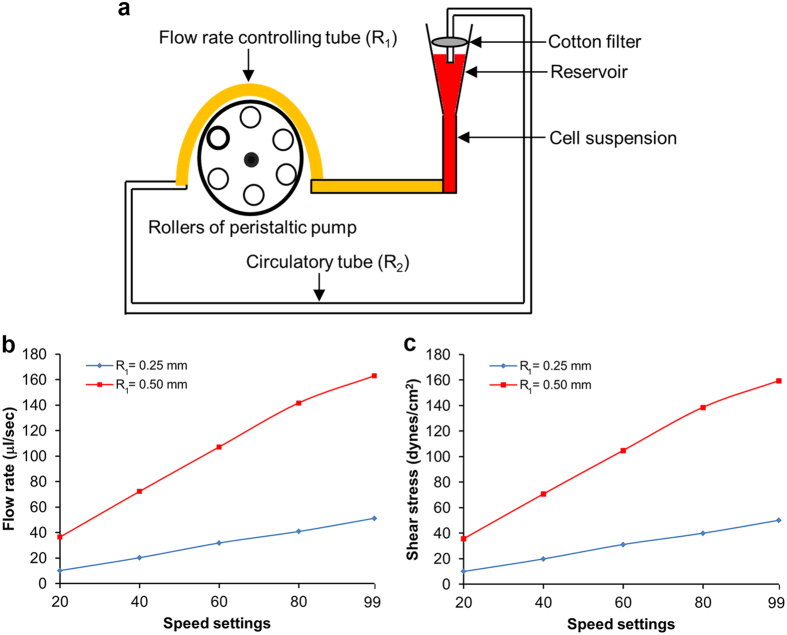
Design of the microfluidic circulatory system. (**a**) Schematic diagram of the microfluidic circulatory system that can generate various levels of SS. Correlations between the speed settings of the peristaltic pump with the flow rate (**b**) and levels of SS (**c**).

A microfluidic circulatory system was developed based on our previous work^18^ to study the effects of hemodynamic SS on CTCs (Fig. 1a). This system can generate various levels of SS that CTCs may encounter in the human vascular system under both resting and intensive exercise conditions. This circulatory system consists of four parts: 1) a reservoir for loading the cell suspension into the system that also allows oxygen and carbon dioxide to get into the tubing system. To ensure the culturing condition of the circulatory system is similar to that of the incubator, we have put the whole system including the pump into the CO_2_ incubator and maintained the whole system within the incubator during the entire circulation time; 2) a cotton filter for preventing both airborne contamination and evaporation of the culture medium in the reservoir; 3) a durable controlling tube (PharMed^®^) that contacts the six rollers of the peristaltic pump to control the flow rate (shown in yellow color in Fig. 1a); and 4) a circulatory tube (silicone tubing) that allows cell suspension to flow unidirectionally and return to the reservoir. The rotation of the rollers that contact the durable tube can drive the fluid to flow in a pulsatile manner with adjustable flow rate. The durable tube has a radius R_1_ of either 0.25 mm or 0.5 mm and a fixed length of 30 cm, whereas the circulatory tube has a radius R_2_ of 0.25 mm and a length of 1.5 m.

The highest SS used in our previous study was 30 dynes/cm^2^ which is two times higher than the average artery SS^12^,^18^,^19^. In order to produce a higher level of SS (60 dynes/cm^2^) that can be generated in the femoral artery during intensive arm cycle exercise^20^, we first determined the correlations between the pump speed and SS by using the same radius (R = 0.25 mm) for both controlling (R_1_) and circulatory (R_2_) tubes. The flow rate (Q) in ml/sec was calculated by measuring the volume of culture medium that exited the circulatory tube in 30 sec at each tested speed (Fig. 1b). With the measured flow rate, the fluid SS can be calculated using Poiseuille’s equation: 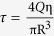, where η is the fluidic dynamic viscosity which is 0.012 dynes•sec/cm^2^. At the fastest speed setting of 99, the highest SS of this microfluidic circulatory system was 50 dynes/cm^2^ (Fig. 1c). To increase the SS, we doubled the radius of the flow rate-controlling tube to R_1_ = 0.50 mm; this tube was connected to the circulatory tube with the same radius of R_2_ = 0.25 mm used in the previous experiment. As a result, the flow rate was increased (Fig. 1b), which in turn significantly elevated the maximum SS of the system from 50 to 160 dynes/cm^2^ (Fig. 1c). Therefore, we have developed a microfluidic circulatory system that can generate a range of physiologically and exercise relevant shear stresses, including 15, 30, 45 and 60 dynes/cm^2^.

### High shear force can kill CTCs

**Figure 2:**
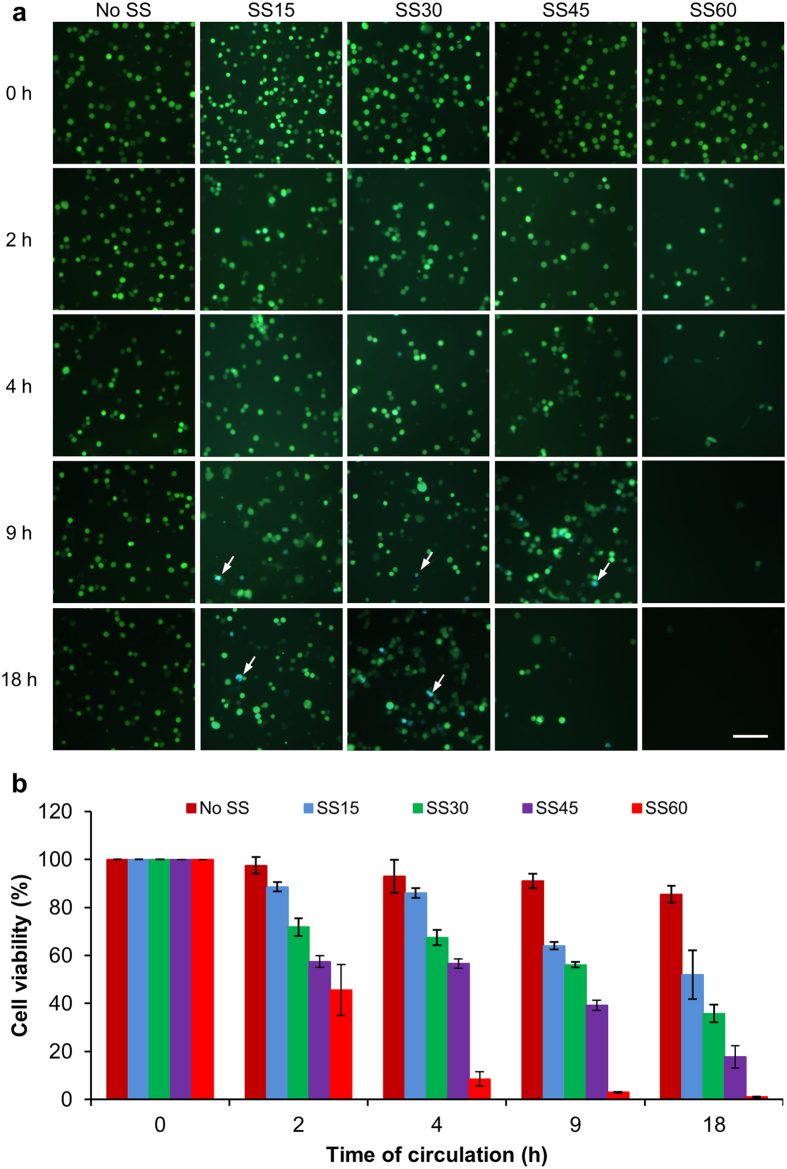
Shear force- and time-dependent death of 231-C3 cells. (**a**) Merged FRET images of 231-C3 cells immediately after being treated with No SS, SS15, SS30, SS45 and SS60 for 0, 2, 4, 9 and 18 h. The white arrows indicate the blue apoptotic cells. Scale bar: 100 μm. (**b**) Viability of 231-C3 cells was measured by the MTT assay under the same conditions indicated in panel (**a**). The results were normalized to 0 h.

To investigate the effects of SS on inducing apoptosis in CTCs, we utilized engineered human breast cancer MDA-MB-231 cells (231-C3) that express apoptotic sensor for real-time detection of caspase activation^22^. Four SS conditions were selected in this experiment: 15, 30, 45, and 60 dynes/cm^2^ covering the range of arterial SS from physiologically resting state^19^ to heavy exercise condition^20^. After 231-C3 cells were circulated in the microfluidic system for 0, 2, 4, 9, and 18 h, the cells were collected from the end of the tube and subjected to FRET imaging analysis. The fluorescent micrographs showed that many 231-C3 cells were still alive after being circulated under lower SS conditions of SS15 and SS30 for 18 h, while very fewer cells survived the higher SS treatment of SS45 for 18 h (Fig. 2a). No viable cells were detected under the highest SS60 treatment for 9 and 18 h, whereas some cells survived this harsh circulatory condition at 2 and 4 h (Fig. 2a). These results indicate that high SS can kill more CTCs than low SS within the same duration of circulatory treatment.

We further validated this finding by measuring the cell viability using the 3-(4,5-dimethylthiazol-2-yl)-2,5-diphenyltetrazolium bromide (MTT) assay. The MTT results showed that 52% and 36% of cells were viable after being circulated under lower levels of SS of 15 and 30 dynes/cm^2^ for 18 h (Fig. 2b). But much lower cell viability of 18% and 1% were detected from the cells treated with higher SS of 45 and 60 dynes/cm^2^ (Fig. 2b). These results confirm the finding that high SS kills more CTCs than low SS within the same duration.

**Figure 3:**
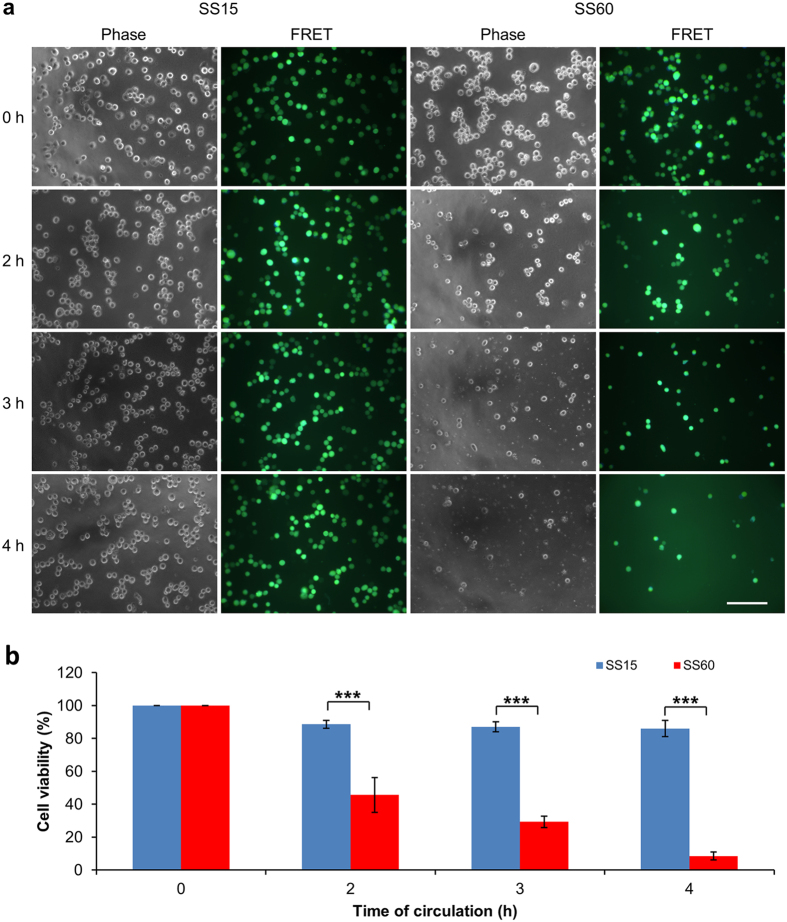
Examination of the SS effects. (**a**) Phase and merged FRET images of the 231-C3 cells after 0, 2, 3 and 4 h of circulation under the treatment of SS15 and SS60. Scale bar: 100 μm. (**b**) Viability of 231-C3 cells was measured by the MTT assay under the same treatment. The results were normalized to 0 h. ***p < 0.001.

To more closely examine the time frame of high SS-induced cell death, the 231-C3 cells were circulated in our microfluidic system under SS15 and SS60 for 2, 3 and 4 h and analyzed by FRET/light imaging microscopy and MTT assay. Both the FRET and phase images showed that high SS60 treatment significantly reduced the number of live cells at 2–3 h and abolished most of the cells at 4 h (Fig. 3a), meanwhile more cell debris could be seen in the phase images of 3–4 h. The results of MTT assay (Fig. 3b) also showed that the cell viability was decreased to 45.6%, 29.3% and 8.5% after high SS treatment of 60 dynes/cm^2^ for 2, 3 and 4 h, respectively. While the cell viability of SS15-treated cells was found to be 86% after 4 h of circulation. These results indicate that 4 h of high SS60 circulation is enough to destroy most of the CTCs, while low SS15 treatment is not effective to kill the CTCs within 4 h.

### High SS can induce necrosis in CTCs

**Figure 4:**
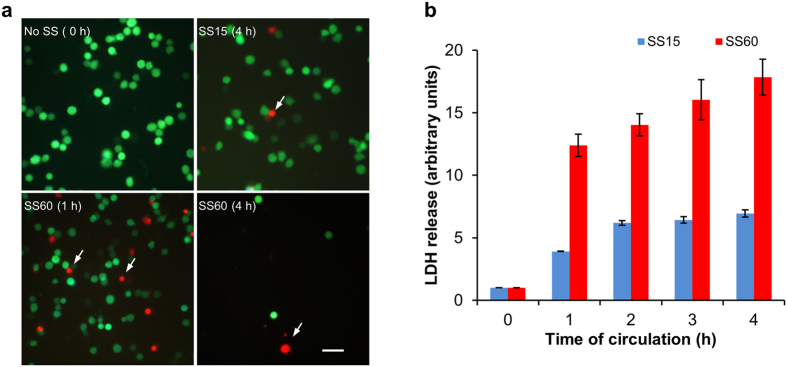
Examination of the necrotic effects of high SS on circulated 231-C3 cells. (**a**) Optical micrograph of 231-C3 cells that were circulated under SS15 and SS60 for 4 h. Necrotic cells were visualized using propidium iodide (PI) staining, while apoptotic cells were indicated by FRET imaging. Necrotic cells emit a red color, whereas the live cells emit a green color. The white arrows indicate the red necrotic cells. Scale bar: 100 μm. (**b**) Relation of LDH release with the time (1–4 h) of SS treatment. The results were normalized to 0 h.

Because the cell viability was drastically reduced from 100% to 8.5% within the first 4 h of high SS treatment, we hypothesized that most of the cells might be killed by necrosis (unregulated cell death) rather than apoptosis (programmed cell death). We tested this hypothesis by using a previously described fluorescence staining method^24^. 231-C3 cells were circulated in our microfluidic system under SS15 or SS60 for 4 h. Then, the cells were collected from the ends of the tubing and added into a 96-well plate. Afterwards, cells were stained with 10 μM propidium iodide (PI) for 30 min. Cells are considered necrotic if their cell membranes are damaged which allows a membrane impermeable dye, such as PI, to enter the cells. The fluorescence images of the PI staining and FRET microscopy were obtained separately and merged to produce Fig. 4a. The fluorescent micrographs showed that high SS of SS60 ruptured the plasma membrane of 231-C3 cells showing positive for PI staining.

To validate the necrotic effect of high SS on CTCs, we performed a lactate dehydrogenase (LDH) assay. LDH is a cytoplasmic enzyme which can be released from the cells into extracellular environment when cell membrane is damaged^25^,^26^. Thus LDH assay can be used to detect necrotic cell death. The assay is based on the reduction of nicotinamide adenine dinucleotide (NAD) by LDH which is then utilized in the stoichiometric conversion of a tetrazolium dye. After circulated at SS15 or SS60 for 1–4 h, the cell suspension was centrifuged and the supernatant was used to perform the LDH assay. The experimental results showed that the level of LDH was significantly increased by ~12 fold after 1 h of high SS60 treatment compared with the uncirculated control cells, and the LDH level was further increased to ~17 fold after 4 h of SS60 treatment. In contrast, much lower levels of LDH release were detected from the cells circulated under low SS15 treatment for 1–4 h (Fig. 4b). This result verifies that high SS can induce necrosis in CTCs.

### High SS prevents CTCs to attach and induces apoptosis after circulation

**Figure 5:**
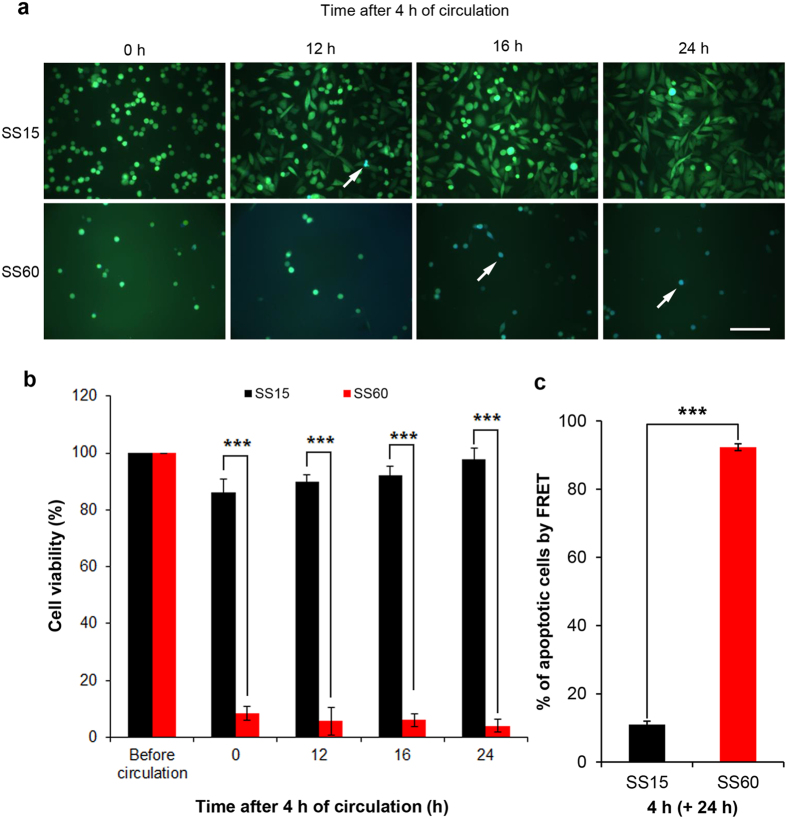
Cell fate after exposure to low and high SS. The cells were exposed to SS15 and SS60 for 4 h and allowed to recover under a static condition. The images were captured during the post-circulation period at the indicated times. (**a**) Merged FRET images of the 231-C3 cells at 12, 16 and 24 h post-circulation under SS15 and SS60 for 4 h. The white arrows indicate the blue apoptotic cells. Scale bar: 100 μm. (**b**) The viability of the 231-C3 cells was measured by the MTT assay under the same treatment conditions as in (**a**). The results were normalized to before circulation. (**c**) The percentage of apoptotic cells was quantified from the FRET images (n = 300 cells, ***p < 0.001).

We next compared the impacts of low SS vs. high SS on the cell recovery during post-circulation time. 231-C3 cells were exposed to SS15 or SS60 for 4 h and allowed to recover for 12, 16, and 24 h in 96-well plates. The FRET images show that after 4 h of SS15 treatment, many live green cells are still visible. Cells started to attach to a normal 96-well plastic culture plate at 12 h and most of them became fully attached at 24 h. Importantly, very few blue apoptotic cells appeared in the FRET images of SS15-treated cells, and quantitative analysis of the FRET images also showed that apoptotic rate of SS15-treated cells was 11% at 24 h of post-circulation (Fig. 5a,c). This result indicates that exposure to low SS of 15 dynes/cm^2^ did not affect cell attachment or induce apoptosis. In contrast, the cells that were treated with high SS of 60 dynes/cm^2^ for 4 h could not attach to the plate after 12 h of incubation. More significantly, most of these cells changed their color from green to blue at 16 h, and 92% of them underwent apoptosis at 24 h (Fig. 5a,c).

The MTT results confirm the findings of the FRET imaging microscopy. The graph in Fig. 5b shows that after circulated at SS15 for 4 h, about 90% of the cells were alive at 12 h post-circulation and even higher viability of 96.7% were registered at 24 h post-circulation, probably due to the growth of cells after the recovery (Fig. 5b). However, circulating cells at high SS60 for 4 h reduced cell viability to 8.5%, which was further reduced to 4% at 24 h post-circulation (Fig. 5b).

### High SS can effectively kill other types of cancer cells

**Figure 6:**
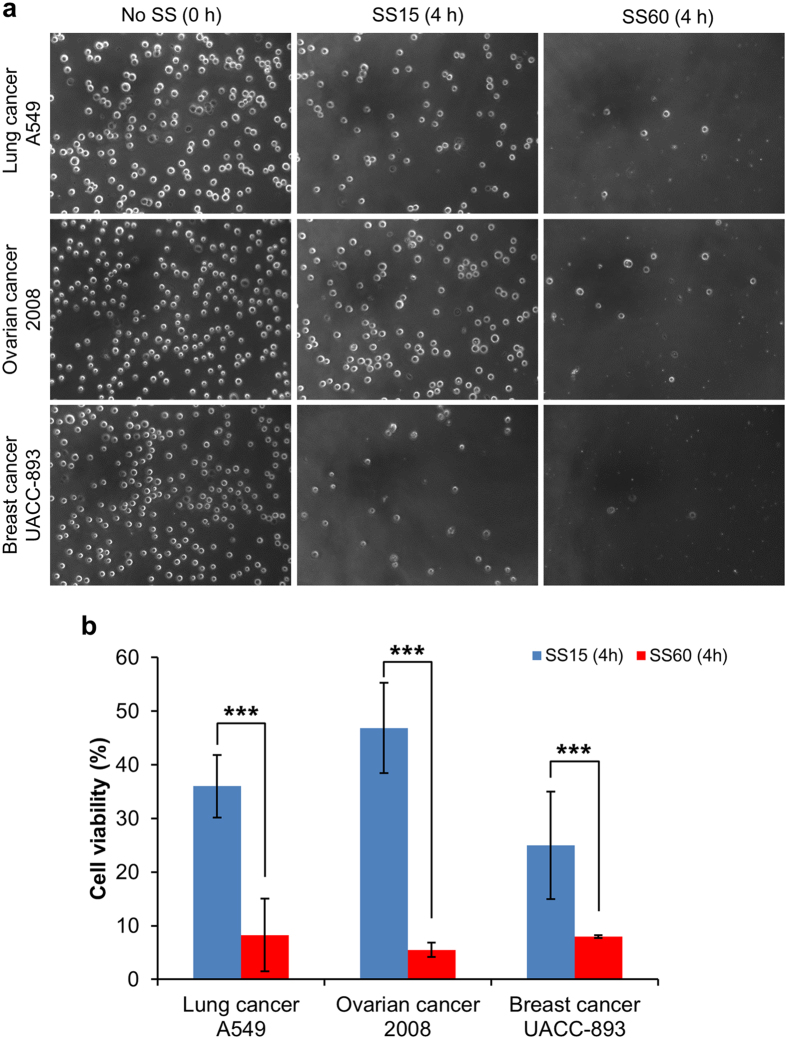
Examining the cytotoxic effects of SS on multiple types of cancer cells. (**a**) Optical micrographs showing the effects of the low and high SS conditions on three cell lines of A549, 2008 and UACC-893. (**b**) The cell viability was measured by the MTT assay after 4 h of circulation under SS15 and SS60. ***p < 0.001.

To explore the possibility of using high SS as a strategy to kill CTCs in circulation, we evaluated its destructive effect on three other cancer cell lines including: 1) Lung cancer cell line (A549), 2) Ovarian cancer cell line (2008), and 3) another cell line of breast cancer origin (UACC-893). All three types of cells were circulated in our microfluidic system under the SS15 and SS60 conditions for 4 h. The physical presence of the cells after the circulatory treatment was examined by phase-contrast light microscopy. The micrographs in Fig. 6a shows that very similar to the case of 231-C3 cells, very few numbers of cells remained intact after the treatment of SS60 for 4 h. As these cells do not have FRET sensor, we used MTT assay to quantify the cell viability. The MTT results show that less than 8% of the cells survived the high SS treatment, while much higher levels of viability were detected from all three types of cells under a low level of SS15 treatment. These results show that high SS is an effective approach to kill various types of CTCs.

### High SS can also destroy highly metastatic and drug resistant breast cancer cells

**Figure 7:**
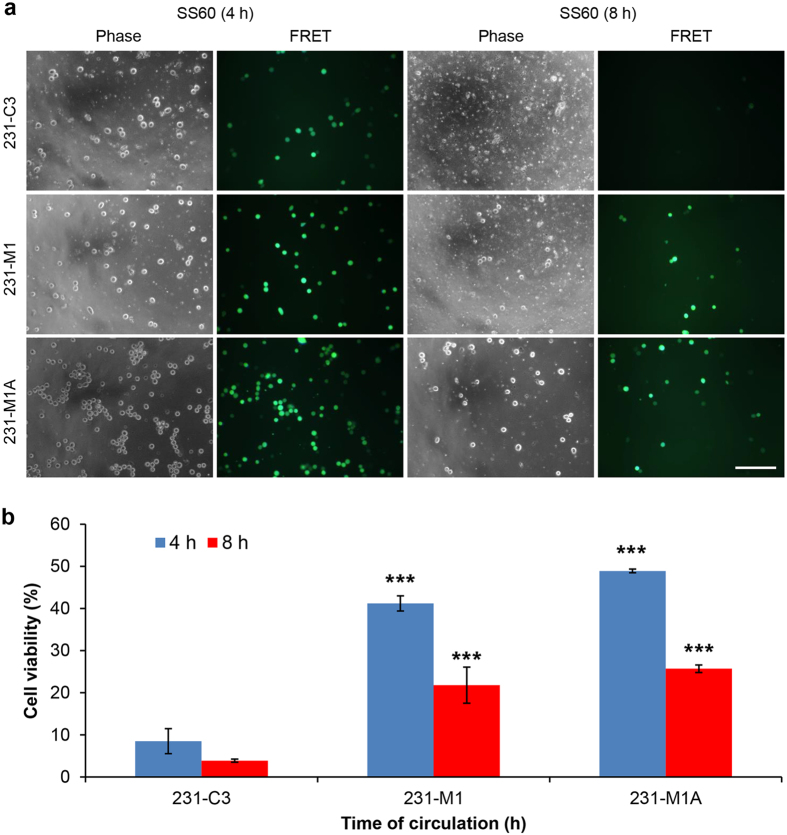
Effects of high SS treatments on cancer cells with different metastatic and drug resistant abilities. (**a**) Merged FRET images of 231-C3, 231-M1 and 231-M1A cells after 4 and 8 h of SS60 treatment. Scale bar: 100 μm. (**b**) Viability of 231-series cells was measured by the MTT assay at 4 and 8 h after SS60 treatment. The results were normalized to 0 h. ***p < 0.001 *vs.* 231-C3 cells.

To test whether high SS treatment can be used as a strategy to eliminate highly metastatic CTCs, we utilized a family of 231-C3 cells with increased metastatic ability and resistance to an anticancer drug, doxorubicin^18^. The 231-M1 cells were isolated from a tiny lung tumor after 231-C3 cells were injected into the tail vein of nude mouse for 45 days; and 231-M1A cells were isolated from a much bigger metastatic lung tumor after 231-M1 cells were intravenously injected into the mice for 90 days. Thus, the metastatic capacities of these cell lines should be in the order of 231-M1A > 231-M1 > 231-C3. All three types of cells were circulated in the microfluidic system under the high SS of 60 dynes/cm^2^ for 4 or 8 h. Afterwards, the cells were collected from the tube and analyzed by FRET imaging microscopy. The FRET images showed that although some highly metastatic 231-M1A and 231-M1 cells survived the high SS treatment at 4 h; much less number of these strongly metastatic cells stayed alive after a prolonged high SS treatment for 8 h (Fig. 7a).

We further validated this finding using the MTT assay. The results show that although highly metastatic 231-M1 and 231-M1A cells exhibited stronger resistant than the original 231-C3 cells against the cytotoxic effect of SS60 treatment for 4 h. The viability of these metastatic cells reduced to 22% for 231-M1 cells and 26% for 231-M1A cells after the treatment of SS60 was prolonged to 8 h (Fig. 7b). This result suggests that this physical method may be an effective approach to destroy metastatic and drug resistant cancer cells.

### Leukemic cells are more resistant to high SS than other types of cancer cells

**Figure 8:**
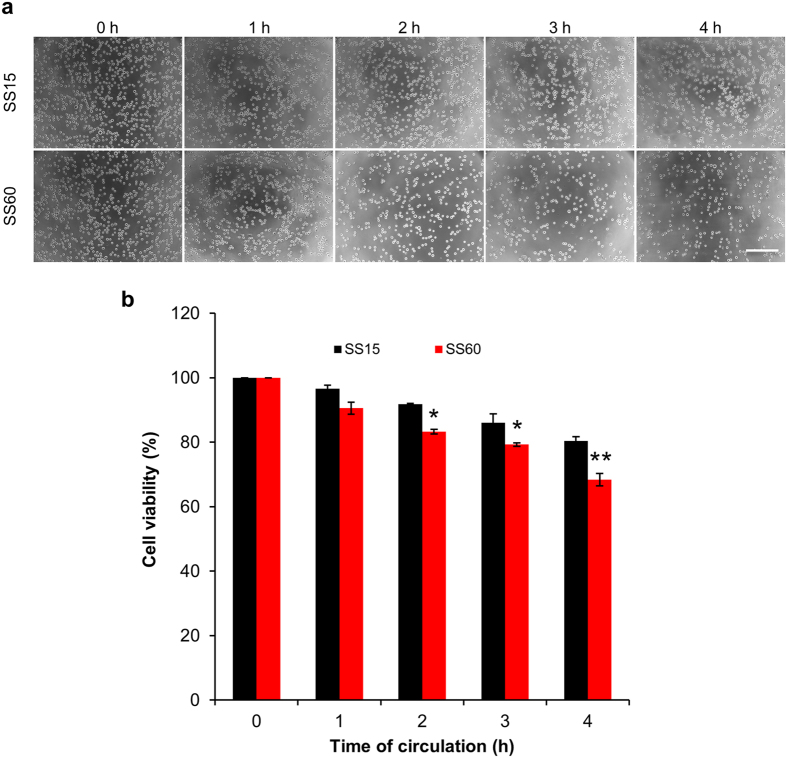
Leukemic cells are highly resistant against strong shear forces. (**a**) Optical micrographs showing the effects of the low and high SS conditions on K562 cells. Scale bar: 200 μm. (**b**) The viability of K562 cells was measured by the MTT assay after 1–4 h of SS15 and SS60 treatment. The results were normalized to 0 h. *p < 0.05, **p < 0.01.

To address the concern that high SS might damage white blood cells while it destroys the CTCs in the bloodstream, we used leukemia K562 as model cells to check their survivability after the circulatory treatments. Cells were circulated in our microfluidic system under the treatments of SS15 and SS60 for 1–4 h, respectively, and analyzed by imaging microscopy and MTT assay. Unlike the breast, lung and ovarian cancer cells, where more than 92% of cells died after circulation under SS60 for 4 h (Fig. 6b); many K562 cells remained intact after 4 h of SS60 treatment (Fig. 8a). We also performed the MTT assay and the results showed that 68.3% of cells were viable under SS60 for 4 h which is slightly lower than the viability (80.4%) of SS15 at 4 h (Fig. 8b). As SS15 is the average arterial SS, while SS60 represent a higher SS achievable during arm cycle exercise, we predict that applying a high SS60 for 4 h can selectively damage CTCs without significantly affecting the white blood cells in the circulatory system.

## Discussion

Previous studies on hemodynamic SS mainly focused on its role in the structure and function of endothelial cells and its involvement in cardiovascular diseases^16^,^27^,^28^. Separate studies also focused on the isolation of CTCs^29^. However, few studies investigated the destructive effects of SS on CTCs, whose survival is a prerequisite for metastasis. Many tumor cells can enter the circulatory system during metastasis; however, only the survived CTCs can reach the metastatic sites and grow into secondary tumors in distinct organs of the body^2^,^3^,^4^. If we can understand how CTCs withstand the fluid shear force, we can develop new strategy to specifically destroy CTCs, thus effectively preventing metastasis.

Hemodynamic SS exists everywhere in the circulatory system and every CTC will experience this mechanical force during circulation^19^,^30^. Previously, other studies have used various devices including a cone-plate viscometer and a syringe needle to generate fluid SS to study its damaging effect on cancer cells^19^,^28^,^31^,^32^. Recently, our lab has developed a microfluidic circulatory system that can generate physiologically relevant SS. Using this system, we showed that low SS of 15 dynes/cm^2^ was effective to induce apoptosis in nonmetastatic breast cancer MCF7-C3 cells, but did not cause significant cell death against metastatic breast cancer 231-C3 cells^18^. In this study, we modified this microfluidic system to generate higher levels of SS that can be produced in the femoral artery during intensive exercise^19^,^20^.

Using this system, we found that circulation under high SS of 60 dynes/cm^2^ for 4 h could kill 90% of the CTCs derived from multiple types of cancer including breast cancer MDA-MB-231, UACC-893, lung cancer A549, and ovarian cancer 2008. High SS killed CTCs through two mechanisms: first via necrosis within the 4 h of circulatory treatment, and second via apoptosis during the 24 h of post-circulatory incubation. Furthermore, circulation under SS60 for 8 h could destroy 74% of metastatic breast cancer cells (231-M1A) that were found to be highly malignant and strongly resistant to doxorubicin in our previous study^18^. Interestingly, leukemia K562 cells representing the white blood cells showed strong resistance to the high SS treatment.

Based on these findings, we speculate that cancer patients may use exercise as a mean to produce high SS which in turn can kill CTCs in their vascular system. This physical approach shall have at least three advantages over chemo- and radio-therapies: (1) it has higher specificity on CTCs than the surrounding blood cells; (2) it has stronger efficacy against metastatic and drug resistant CTCs; and (3) it should have much less side effects to the cancer patients.

We are currently investigating the mechanisms by which high SS induces cell death. There are two possibilities. First, we have observed in this study that the cells that were circulated under SS60 for 4 h could not attach to the surface after 12 h of incubation and underwent apoptosis 16–24 h later (Fig. 5a). It is possible that the circulatory treatment at high SS damaged the cytoskeleton, prevented cell adhesion, and induced apoptosis via anoikis. However, anoikis is unlikely to be the major cause of SS-induced cell death because we observed that circulating CTCs under low SS15 for 18 h caused only 48% of cell death (Fig. 2b). The second possible cause of cell death is related to our previous finding that SS could elevate the level of reactive oxygen species (ROS) in endothelial cells^15^ and CTCs^18^. ROS may also be elevated in CTCs in response to high SS, which can thus trigger oxidative stress-induced cell death.

## Materials and Methods

### Cell lines and cell culture

Breast cancer MDA-MB-231 was provided by Prof. Xiao-Feng Le from the Department of Experimental Therapeutics at the University of Texas M.D. Anderson Cancer Center, Houston, TX, USA. The fluorescence resonance energy transfer (FRET)-based caspase sensor C3^23^ was transfected into MDA-MB-231 cells to generate a stable cell line named 231-C3^22^. The 231-C3 cells can express a CFP-DEVD-YFP fusion protein, which can be cleaved by caspase-3 or caspase-7, thus serving as a live indicator of apoptosis. A family of 231-C3 cells with increased metastatic ability were generated by injecting 231-C3 cells into the tail vein of nude mice. Afterwards, metastatic sensor cells were isolated from lung metastases in two rounds of such experiments^18^. Other cell lines including lung cancer A549, ovarian cancer 2008, breast cancer UACC-893, and leukemia K562 cells were obtained from ATCC.

231-C3, 231-M1, 231-M1A, and UACC-893 cells were cultured in Dulbecco’s modified Eagle’s medium (DMEM, Invitrogen, USA). A549, 2008, and K562 cells were cultured in Roswell Park Memorial Institute (RPMI 1640, (Invitrogen, USA). All the culture media were supplemented with 10% (v/v) fetal bovine serum (FBS, Hyclone, USA), 100 units/ml penicillin, and 100 mg/ml of streptomycin (Gibco, USA). Cells were cultured in a 37 °C incubator with 5% CO_2_.

### Microfluidic circulatory system

A microfluidic circulatory system with a peristaltic pump (Ismatec, Germany) was constructed to control the flow rate of the system. Our microfluidic system is based on 6 rollers (Fig. 1a) which pumps the fluid in a pulsatile manner mimicking the flow of bloodstream in the human body^21^. DMEM or RPMI 1640 containing 10% FBS, 1% penicillin and streptomycin, pH 7.4 (PS, Gibco, USA) was used as fluid composition in the circulating system. The SS that the cells encounter in the circulation can be calculated using Poiseuille’s equation^33^: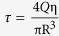, where Q is the flow rate in ml/sec, η is the dynamic viscosity of the fluid, which equals to 0.012 dynes.sec/cm^2^, and R is the inner radius of the circulatory tube (R_2_). We have tested various cell densities ranging from 1 × 10^4^ to 1 × 10^6^ cells/ml and found that 2 × 10^5^ cells/ml is the best condition at which enough cells can be collected for analysis, and meanwhile they can circulate in the system without conjugation or precipitation. The length of the silicone-based circulatory tube (Cole Parmer, USA) is 1.5 m; the size of R_1_ is either 0.25 mm or 0.5 mm, and the size of R_2_ is fixed at 0.25 mm (Fig. 1a). The tube was sterilized by washing with 70% ethanol (4 ml), followed by rinsing with autoclaved distilled water (4 ml). To reduce the adhesion of the circulating cells to the tube, the inner surface of the tube was treated with 1% Pluronic F-127 (Invitrogen, USA) for 30 min. Then, the Pluronic F-127 was removed, and the tube was air dried for at least 30 min. The morphology of cells is examined every time before the cells were introduced into the microfluidic system to make sure that only healthy cells were used. The cells were trypsinized and suspended in fresh DMEM at a density of 2 × 10^5^/ml. One milliliter of the cell suspension (2 × 10^5 ^cells) was injected into the circulatory system and subjected to SS treatment for various durations at 37 °C in a 5% CO_2_ incubator. The same volume (100 μl) of cell suspension were then collected at the designated time points.

### Fluorescence resonance energy transfer (FRET) imaging microscopy

The FRET images were captured using an inverted Zeiss Axiovert S100 fluorescence microscope (Carl Zeiss, Germany). The cells were excited with a light at the wavelength of 436 ± 10 nm. The emission fluorescent images of cyan fluorescent protein (CFP) (480 ± 20 nm) and yellow fluorescent protein (YFP) (535 ± 15 nm) were captured using a computer-controlled charge-coupled device (CCD) camera (AxioCam MRm, Carl Zeiss, Germany). The digital fluorescence images of CFP and YFP were merged using Image-Pro Plus software (Media Cybernetics, Inc. USA). The merged FRET images were further analyzed to determine the percentage of apoptotic cells. The live cells appeared in green in the merged FRET image. When cells undergo apoptosis, caspase-3 is activated, which leads to the cleavage of the peptide linker at the DEVD sequence between CFP and YFP. This cleavage disrupts the energy transfer from CFP to YFP. As a result, the FRET image changes from green to blue, indicating the occurrence of apoptosis. The rate of apoptosis is calculated using the following formula:


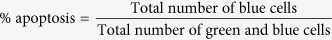


### MTT assay

MTT assay can measure the level of reductase in mitochondria which reflects the level of cell metabolic activity. In this assay, the activity of NAD(P)H-dependent cellular oxidoreductase represents the total number of viable cells. This enzyme basically reduces the MTT 3-(4,5-dimethylthiazol-2-yl)-2,5-diphenyltetrazolium bromide into formazan (insoluble). After various SS treatments, the cells were collected from the circulatory system and 100 μl of suspended cells were added to each well of a 96-well plate. After various incubation times, 10 μl of sterilized MTT solution (5 mg/ml) was added to the cells. After 3 h incubation, 100 μl of solubilization solution containing 10% SDS and 0.1% HCl was added to each well. 12 h later, the optical density was measured at 595 nm using a plate reader (PerkinElmer Victor^3^).

### Detection of necrosis by propidium iodide (PI) staining

Propidium iodide (PI) can penetrate damaged cell membranes and stain the nucleus red; thus, PI staining has been used as the conventional assay for determining necrotic cell death. One milliliter of 2 × 10^5^ cells was injected into the microfluidic system. In this study, after different SS treatment for 1–4 h, the same volume (100 μl) of cell suspension were stained with 10 μM PI (Sigma, USA) for 30 min in the dark. The images were captured with an inverted fluorescence microscope (Axio Observer Z1, Carl Zeiss, Germany) at an excitation wavelength of 590 nm and emission wavelength of 630 nm.

### LDH assay

The lactate dehydrogenase assay was performed using a commercial test kit (Sigma-Aldrich). One milliliter of 2 × 10^5^ cells was injected into the microfluidic system. After various SS treatments, the same volume (100 μl) of cell suspension were collected from the circulatory system and centrifuged at 1500 RPM for 3 min. The cell pellet was discarded and 100 μl of supernatant was transferred to each well of a 96-well plate. The LDH mixture (200 μl/well) was added to the supernatant, immediately after which, the plate was covered with aluminum foil. After 30 min of incubation at room temperature, 30 μl of 1 N HCl was added to each well to terminate the reaction. Then, the primary absorbance and background absorbance was measured at 490 nm and 690 nm respectively using a plate reader (Spectramax M5). The background absorbance at 690 nm was subtracted from the primary absorbance at 490 nm which was then used to calculate the LDH release.

### Statistical analysis

All of the data are presented as the means ± standard deviations from at least three independent experiments. Student’s t-test for two-tailed distributions and two-sample unequal variances were used to calculate the p-values between two groups. *p < 0.05, **p < 0.01, ***p < 0.001 were considered significant. The FRET images were quantified using micrographs obtained from three independent experiments. A total of 300 cells were counted.

## Additional Information

**How to cite this article**: Regmi, S. *et al*. High Shear Stresses under Exercise Condition Destroy Circulating Tumor Cells in a Microfluidic System. *Sci. Rep.* **7**, 39975; doi: 10.1038/srep39975 (2017).

**Publisher's note:** Springer Nature remains neutral with regard to jurisdictional claims in published maps and institutional affiliations.
